# Determination of Predominance of Influenza Virus Strains in the Americas

**DOI:** 10.3201/eid2107.140788

**Published:** 2015-07

**Authors:** Eduardo Azziz-Baumgartner, Rebecca J. Garten, Rakhee Palekar, Mauricio Cerpa, Sara Mirza, Alba Maria Ropero, Francisco S. Palomeque, Ann Moen, Joseph Bresee, Michael Shaw, Marc-Alain Widdowson

**Affiliations:** Centers for Disease Control and Prevention, Atlanta, Georgia, USA (E. Azziz-Baumgartner, R.J. Garten, R. Palekar, S. Mirza, F.S. Palomeque, A. Moen, J. Bresee, M. Shaw, M.-A. Widdowson);; Pan American Health Organization, Washington, DC, USA (R. Palekar, M. Cerpa, A.M. Ropero)

**Keywords:** Influenza, influenza virus, epidemic, timing, strain, vaccine, North America, South America, Central America, viruses

## Abstract

During 2001–2014, predominant influenza A(H1N1) and A(H3N2) strains in South America predominated in all or most subsequent influenza seasons in Central and North America. Predominant A(H1N1) and A(H3N2) strains in North America predominated in most subsequent seasons in Central and South America. Sharing data between these subregions may improve influenza season preparedness.

During 2002–2008, infection with influenza viruses caused 40,880–160,270 deaths each year throughout the Americas (*1*). To prevent illness and death, medical staff in 35 countries throughout the Americas administer influenza vaccines (*2*). However, producing the vaccine takes ≈6 months, and selecting virus strains necessitates assessing which strains are likely to predominate during upcoming epidemics (*3*).

Surveillance for influenza has improved dramatically, especially in the American tropics (*4*). Nevertheless, it remains unclear whether virus strains identified in North America subsequently become predominant in South America and vice versa (*3*). Such information could help public health officials in each hemisphere prepare for upcoming influenza seasons. We describe influenza epidemics in North, Central, and South America and explore whether the virus strains that caused them were similar.

## The Study

We obtained the number of respiratory swabs tested throughout each year and the number that were positive for influenza virus from the Global Influenza Surveillance and Response System (*5*). Data from Canada, Mexico, and the United States (population 458 million) collected during 2002–2013 were aggregated to represent North America; data from Belize, Costa Rica, El Salvador, Guatemala, Honduras, Nicaragua, and Panama (population 42 million) to represent Central America; and data from Argentina, Brazil, Chile, Paraguay, and Uruguay (population 262 million) to represent South America (*6*). We obtained antigenic characterization data from the Centers for Disease Control and Prevention (Atlanta, GA, USA).

We determined the proportion of respiratory specimens that tested positive for influenza virus each month in North, Central, and South America and then determined the annual median for each subregion; months in which the proportion exceeded the annual median were considered epidemic (*7*). The timing and length of epidemics in each subregion were also explored, and the proportion of samples testing positive for influenza virus was used as a proxy for epidemic severity. Antigenic virus strains were defined as predominant if they made up the largest proportion of positive samples by type or subtype during an influenza season.

We assessed whether predominant virus strains identified in South America were subsequently identified in Central and North America and whether strains identified in North America were subsequently identified in Central and South America. We also investigated whether predominant strains were represented by components of available Southern or Northern Hemisphere vaccine formulations.

During 2002–2013, South America reported 877,770 influenza-positive respiratory samples (2.8/10,000 persons/y) and North America 4,535,508 results (9.0/10,000 persons/y) to the Global Influenza Surveillance and Response System (*5*). During 2006–2013, Central America reported 82,163 results (2.4/10,000 persons/y). In each subregion, the number of reports increased during the study period (p = 0.02). During 2006–2013, the Centers for Disease Control and Prevention analyzed 2,971 samples from South America, 1,279 from Central America, and 215,127 from North America for antigenic characterization.

In South America, influenza epidemics started in April, in Central America in June, and in North America in December. With the exception of 2 (25%) of 8 years in Central America and 2 (17%) of 12 years in South America, when there was 1 southern temperate winter epidemic and a smaller northern temperate winter epidemic, all subregions had 1 annual influenza epidemic that lasted ≈5 months.

The predominant influenza A(H1N1) virus strains in South America predominated in 9 of 9 subsequent seasons in Central America and 12 (92%, 95% CI 78%–107%) of 13 subsequent seasons in North America (Table 1; Technical Appendix). Similarly, A(H3N2) virus strains in South America predominated in 11 (92%, 95% CI 76%–107%) of 12 subsequent seasons in Central America and 10 (71%, 95% CI 48%–95%) of 11 subsequent seasons in North America. Predominant influenza B virus strains in South America only predominated in 8 (67%, 95% CI 40%–93%) of 12 subsequent seasons in Central America and 8 (57%, 95% CI 31%–83%) of 14 subsequent seasons in North America. The proportion of influenza B virus strains in South America that predominated in subsequent seasons in North America, however, increased from 55% to 73% when we accounted for all identified virus strains and not just those that predominated in South America. Virus strains in South America during 1 season typically did not predominate in subsequent seasons in South America (54%, 95% CI 38%–70%).

**Table 1:** Most commonly identified antigenic characterizations of influenza strains in the Americas during 2002–2013*†

Year	Influenza A(H1N1) virus				Influenza A(H3N2) virus				Influenza B virus		
	South	Central	North		South	Central	North		South	Central	North
2001	A	NA	A		A	NA	A		A	NA	B‡
2002	A	NA	A		A	NA	A		B	B	B
2003	A	NA	A		A	B	B‡		B	NA	C‡
2004	NA	NA	A		B	B	C‡		C	C	C
2005	A	NA	A		C	C	C		D‡	E‡	D
2006	A	A	A		D‡	D	D		E	D/E	D
2007	A	A	B‡		E‡	E	E		F‡	F	F
2008	C‡	C	C		E	E	E		F	F	G‡
2009	D‡	D	D		E	E	F‡		F	G	G
2010	D	D	D		F	F	F		G	G	G
2011	D	D	D		F	F	F		G	G	G
2012	D	D	D		F	F	F		H‡	G	H
2013	D	D	D		G‡	G	H‡		G	I‡	I
2014	D	D	D		H	H	H		I	I	I

*Data from Canada, Mexico, and the United States were aggregated to represent North America; data from Belize, Costa Rica, El Salvador, Guatemala, Honduras, Nicaragua, and Panama to represent Central America; and data from Argentina, Brazil, Chile, Paraguay, and Uruguay to represent South America. †For influenza A(H1N1) virus, A, A/New Caledonia/20/99(H1N1); B, A/Solomon Islands/03/2006(H1N1); C, A/Brisbane/59/2007(H1N1); D, A/California/07/2009-(H1N1)pdm09. For influenza A(H3N2) virus, A, A/Panama/2007/99(H3N2); B, A/Fujian/411/2002(H3); C, A/California/07/2004(H3N2); D, A/Wisconsin/67/2005(H3N2); E, A/Brisbane/10/2007(H3N2); F, A/Perth/16/2009(H3N2); G, A/Victoria/361/2011(H3N2); H, A/Texas/50/2012(H3N2). For influenza B virus, A, B/Sichuan/379/99(YAM); B, B/Shandong/7/97(VIC); C, B/Shanghai/361/2002(YAM); D, B/Malaysia/2505/2005(VIC); E, B/Florida/07/2004(YAM); F, B/Florida/04/2006(YAM); G, B/Brisbane/60/2008(VIC); H, B/Wisconsin/01/2010(YAM) I, B/Massachusetts/02/2012(YAM). NA, not applicable. ‡Newly identified strain (8 in South America, 2 in Central America, and 8 in North America).

The predominant A(H1N1) virus strains in North America predominated in 7 (78%, 95% CI 51%–105%) of 9 subsequent seasons in Central America and 10 (83%, 95% CI 62%–104%) of 12 subsequent seasons in South America. A(H3N2) virus strains in North America predominated in 8 (67%, 95% CI 40%–93%) of 12 subsequent seasons in Central America and 10 (77%, 95% CI 54%–100%) of 13 subsequent seasons in South America. Influenza B virus strains in North America predominated in 9 (75%, 95% CI 51%–100%) of 12 subsequent seasons in Central America and 7 (54%, 95% CI 27%–81%) of 13 subsequent seasons in South America. Virus strains that predominated in North America during 1 season were less likely to predominate in the subsequent season in North America (62%, 95% CI 46%–77%).

At least 1 component of the Southern Hemisphere vaccine composition recommendations matched a predominant antigenic characterization in South America in 13 (93%, 95% CI 79%–106% of 14 influenza seasons that occurred during 2001–2014, and at least 1 component of the Northern Hemisphere vaccine composition recommendations matched a predominant antigenic characterization in North America in all 14 influenza seasons that occurred during 2001–2014. Of 33 predominant antigenic virus strains identified in Central America during 2002–2014, 21 (64%, 95% CI 47%–80%) matched the Southern Hemisphere recommendations and 24 (73%, 95% CI 58%–88%) matched the Northern Hemisphere recommendations (Table 2).

**Table 2:** Most commonly identified antigenic characterizations of influenza strains in Central America compared with composition of Southern and Northern hemisphere vaccines, 2006–2013*

Predominant strains in Central America	South America vaccine composition	Matched southern vaccine	North America vaccine composition	Matched northern vaccine
2002				
	A/New Caledonia/20/99(H1N1)-like	NA	A/New Caledonia/20/99(H1N1)-like	NA
	A/Moscow/10/99(H3N2)-like	NA	A/Moscow/10/99(H3N2)-like	NA
B/Shandong/7/97(VIC)	B/Sichuan/379/99-like	–	B/Hong Kong/330/2001-like	–
2003				
A/Fujian/411/2002(H3)-like	A/New Caledonia/20/99(H1N1)-like	NA	A/New Caledonia/20/99(H1N1)-like	NA
	A/Moscow/10/99(H3N2)-like	–	A/Moscow/10/99(H3N2)-like	–
	B/Hong Kong/330/2001-like	NA	B/Hong Kong/330/2001-like	NA
2004				
	A/New Caledonia/20/99(H1N1)-like	NA	A/New Caledonia/20/99(H1N1)-like	NA
A/Fujian/411/2002(H3)-like	A/Fujian/411/2002(H3N2)-like	+	A/Fujian/411/2002(H3N2)-like	+
B/Shanghai/361/2002(YAM)	B/Hong Kong/330/2001-like	–	B/Shanghai/361/2002-like	+
2005				
	A/New Caledonia/20/99(H1N1)-like	NA	A/New Caledonia/20/99(H1N1)-like	NA
A/California/07/2004(H3N2)-like	A/Wellington/1/2004(H3N2)-like	–	A/California/7/2004(H3N2)-like	+
B/Florida/07/2004(YAM)	B/Shanghai/361/2002-like	–	B/Shanghai/361/2002-like	–
2006				
A/New Caledonia/20/99(H1N1)-like	A/New Caledonia/20/99(H1N1)-like	+	A/New Caledonia/20/99(H1N1)-like	+
A/Wisconsin/67/2005 (H3N2)-like	A/California/7/2004(H3N2)-like	–	A/Wisconsin/67/2005 (H3N2)-like	+
B/Malaysia/2505/2005(VIC)	B/Malaysia/2506/2004-like	+	B/Malaysia/2506/2004-like	+
2007				
A/New Caledonia/20/99(H1N1)-like	A/New Caledonia/20/99(H1N1)-like	+	A/Solomon Is/3/2006 (H1N1)-like	–
A/Brisbane/10/2007 (H3N2)-like	A/Wisconsin/67/2005(H3N2)-like	–	A/Wisconsin/67/2005 (H3N2)-like	–
B/Florida/04/2006(YAM)	B/Malaysia/2506/2004-like	–	B/Malaysia/2506/2004-like	–
2008				
A/Brisbane/59/2007(H1N1)-like	A/Solomon Is/3/2006(H1N1)-like	–	A/Brisbane/59/2007 (H1N1)-like	+
A/Brisbane/10/2007(H3N2)-like	A/Brisbane/10/2007(H3N2)-like	+	A/Brisbane/10/2007 (H3N2)-like	+
B/Florida/4/2006(YAM)	B/Florida/4/2006-like	+	B/Florida/4/2006-like	+
2009				
A/California/7/2009(H1N1)-like	A/Brisbane/59/2007(H1N1)-like	–	A/Brisbane/59/2007 (H1N1)-like	–
A/Brisbane/10/2007(H3N2)-like	A/Brisbane/10/2007(H3N2)-like	+	A/Brisbane/10/2007 (H3N2)-like	+
B/Brisbane/60/2008(VIC)	B/Florida/4/2006-like	–	B/Brisbane/60/2008-like	+
2010				
A/California/7/2009(H1N1)-like	A/California/7/2009(H1N1)-like	+	A/California/7/2009 (H1N1)-like	+
A/Perth/16/2009(H3N2)-like	A/Perth/16/2009(H3N2)-like	+	A/Perth/16/2009 (H3N2)-like	+
B/Brisbane/60/2008(VIC)	B/Brisbane/60/2008-like	+	B/Brisbane/60/2008-like	+
2011				
A/California/7/2009(H1N1)-like	A/California/7/2009(H1N1)-like	+	A/California/7/2009 (H1N1)-like	+
A/Perth/16/2009(H3N2)-like	A/Perth/16/2009(H3N2)-like	+	A/Perth/16/2009 (H3N2)-like	+
B/Brisbane/60/2008(VIC)	B/Brisbane/60/2008-like	+	B/Brisbane/60/2008-like	+
2012				
A/California/7/2009(H1N1)-like	A/California/7/2009(H1N1)pdm09-like	+	A/California/7/2009(H1N1)pdm09-like	+
A/Perth/16/2009(H3N2)-like	A/Perth/16/2009(H3N2)-like	+	A/Victoria/361/2011(H3N2)-like	–
B/Brisbane/60/2008-like(VIC)	B/Brisbane/60/2008-like	+	B/Wisconsin/1/2010-like	–
2013				
A/California/7/2009(H1N1)-like	A/California/7/2009(H1N1)pdm09-like	+	A/California/7/2009(H1N1)pdm09-like	+
A/Victoria/361/2011(H3N2)-like	A/Victoria/361/2011(H3N2)-like	+	A/Victoria/361/2011	+
B/Massachusetts/02/2012(YAM)	B/Wisconsin/1/2010-like	–	B/Massachusetts/02/2012-like	+
2014				
A/California/07/2009(H1N1)pdm09- like	A/California/7/2009 (H1N1)pdm09-like	+	A/California/7/2009(H1N1)pdm09-like	+
A/Texas/50/2012(H3N2)-like GP	A/Texas/50/2012 (H3N2)-like	+	A/Texas/50/2012(H3N2)-like	+
B/Massachusetts/02/2012(YAM)	B/Massachusetts/2/2012-like	+	B/Massachusetts/2/2012-like	+
Total percentage matching strains		64%		73%

*Values are proportions of occurrences when predominant strains are represented in each vaccine formulation. +, match; –, no match; NA, influenza type not among predominant circulating strains; Solomon Is, Solomon Islands.

## Conclusions

Our findings suggest that virus strains identified during influenza epidemics in South America typically became predominant in subsequent epidemics in Central and North America. Almost as frequently, virus strains identified during epidemics in North America became predominant in the subsequent Central and South America epidemics. Although strain selection for 1 hemisphere’s vaccine formulation typically occurs before influenza activity is widespread in the opposite hemisphere, health officials have an opportunity to anticipate which influenza virus strains may predominate by observing activity in other subregions. For example, influenza A(H1N1)pdm09 virus predominated in Brazil during 2013 (*8*) and became predominant in North America during 2013–2014. Health officials identifying influenza B virus strains in 1 hemisphere would have correctly predicted the predominant influenza B virus strains in the opposite hemisphere only half of the time unless they had also examined other co-circulating influenza B virus strains. Nevertheless, such findings underscore the importance of year-round surveillance, viral characterization, data sharing, and annual influenza vaccination.

Our analyses are based on a convenience sample of respiratory specimens obtained from heterogeneous surveillance systems using different diagnostic assays (e.g., PCR and immunofluorescence) and then aggregated by subregion. These samples may not be geographically representative. Additional data will be needed to determine whether the characteristics of 1 subregion reliably predict influenza epidemics in another. New viral strains that appear might be introduced from outside the Americas (*3*).

In summary, health officials in North and Central America may find clues about which influenza A virus strains are likely to predominate during an upcoming season by observing which were predominant in South America and vice versa. Our findings underscore the need to share timely and representative specimens with World Health Organization Collaborating Centres. In the future, shorter vaccine production times using novel technology might facilitate matching vaccine composition more closely to circulating virus strains.

Technical Appendix Most commonly identified antigenic characterizations of influenza strains in the Americas during 2002–2013.

## References

[1] Cheng PY, Palekar R, Azziz-Baumgartner E, Iuliano D, Alencar AP, Bresee J, Burden of influenza-associated deaths in the Americas, 2002–2008. Influenza Other Respir Viruses. In press 2015. 10.1111/irv.12317 PMC4549098 26256291

[2] Ropero-Álvarez AM, Kurtis HJ, Danovaro-Holliday MC, Ruiz-Matus C, Andrus JK. Expansion of seasonal influenza vaccination in the Americas. BMC Public Health. 2009;9:361. 10.1186/1471-2458-9-361 19778430 PMC2764707

[3] Russell CA, Jones TC, Barr IG, Cox NJ, Garten RJ, Gregory V, et al. Influenza vaccine strain selection and recent studies on the global migration of seasonal influenza viruses. Vaccine. 2008;26(Suppl 4):D31–4. 10.1016/j.vaccine.2008.07.078 19230156

[4] Johnson L, Clará W, Gambhir M, Chacón-Fuentes R, Marín-Correa C, Jara J, Improvements in pandemic preparedness in 8 Central American countries, 2008–2012. BMC Health Serv Res. 2014;14:209. 10.1186/1472-6963-14-209 24886275 PMC4022548

[5] World Health Organization. Global Influenza Surveillance and Response System (GISRS) [cited 2013 May 26]. http://www.who.int/influenza/gisrs_laboratory/en/

[6] United Nations, Department of Economic and Social Affairs. World population prospects: the 2010 revision [cited 2015 May 5]. http://esa.un.org/Wpp/Documentation/WPP%202010%20publications.htm

[7] Azziz Baumgartner E, Dao CN, Nasreen S, Bhuiyan MU, Mah-E-Muneer S, Al Mamun A, et al. Seasonality, timing, and climate drivers of influenza activity worldwide. J Infect Dis. 2012;206:838–46. 10.1093/infdis/jis467 22829641

[8] Secretaria de Vigilância em Saúde. Ministério da Saúde. Influenza: monitoramento até a semana epidemiológica 29 de 2013. Boletim Epidemiológico. 2013;44:1–9.

